# Author Index for Volume 107

**DOI:** 10.1038/bjc.2012.532

**Published:** 2012-12-04

A'Hern, R 1025

Aaltonen, LA 1761

Aarts, MJ 12

Aasebø, U 442

Abajo, A 287

Abdel-Wahab, O 1761

Abe, K 1745

Abkevich, V 1776

Abnet, CC 888

Abraham, I 901

Abramovitch, R 658

Ackland, SP 1525

Acosta, G 1584

Adams, RA 1037

Adenis, A 435

Adjogatse, D 1810

Adke, R 243

Adler, G 435

Adler, K 1525

Aft, RL 7

Agarwal, R 925

Aghajanian, C 1093

Aghcheli, K 888

Agostini, M 977

Agrawal, R 1257

Agur, Z 814

Ahmad, A 585

Ahmad, I 1554

Ahrensberg, J 544

Aieta, M 1286

Aihara, R 31

Aikou, S 275

Ainsworth, P 2005

Aitchison, MA 856

Akbar, NS 1361

Akbari, MR 1783

Åkesson, A 895

Akiyoshi, S 1345

Akslen, LA 1997

Aksoy, S 1815

Albanes, D 1589

Alcindor, T 1864

Aleman, BMP 1637

Alemanno, P 1944

Ali, S 1354

Ali, Z 1664

Alidzanovic, L 961

Alimena, G 904

Al-Kharusi, M 1514

Allen, N 169

Allum, W 1908

Almazán-Moga, A 1374

Aloia, TA 1442

Alonso, M 808

Alsaker, MDK 176

Altman, DG 260

Altundag, K 1815

Alvarez-Flores, MP 977

Amadio, G 37

Amadori, A 1286

Ambros, I 1418

Ambros, P 1418

Amir, O 814

Ammann, RA 234

Amundsen, T 442

Anbalagan, S 291

Anborgh, PH 840

Andersson, S-O 895

Ando, H 31

Ando, M 1239

Andrén, O 895

Andreou, A 1442

Andriole, GL 207

Androulakis, N 1932

Ang, M-K 482

Ang, Y 1125

Ang, YS 1766

Angioli, R 785

Angulo, B 345

Ansari KI 315

Antognoli, A 1302

Antsaklis, A 1869

Aparicio, J 435

Arakawa, K 632

Arcusa, A 1249

Arenas, E 1144

Ariad, S 814

Arkenau, H-T 1025

Arlett, CF 1506

Armel, S 2005

Armesilla, AL 1488

Armstrong, G 585

Armstrong, S 531

Arola, J 1761

Arrigo, A-P 63

Arriola, E 1277

Asao, T 31

Ashor, AE 501

Assadourian, S 598

Astolfi, A 1433

Ataseven, B 1892

Atkin, W 765

Attiyeh, E 1418

Atzori, L 675

Aulitzky, WE 280

Autier, P 1608

Avril, M-F 455

Aylin, P 1213

Aziz, FM 772

Baas, P 161

Baba, H 1233, 1950

Baba, S 1239

Baba, Y 1233, 1950

Baccarani, M 904

Bacolod, MD 1399

Baddeley, J 531

Bae, D-S 91

Baglietto, L 800, 1631

Bago-Horvath, Z 1454

Bai, M 1423

Baio, G 765

Bajdik, CD 800

Baker, JL 165

Baker, L 224

Baker, SD 1100

Bakhanashvili, M 1844

Balabanov, S 1853

Balasingham, S 24

Balks, T 1754

Bamias, A 1869

Ban, J-O 53

Bando, H 340, 1444

Bandres, E 287

Banerji, U 1093, 1797

Bang, Y-J 325

Bangard, C 1840

Bangma, CH 778

Banks, RE 1131

Bapiro, TE 1692

Baracos, VE 931

Barbieri, E 1302

Barcelo, J-R 435

Baren, JP 143

Barile, C 1286

Barnadas, A 1249

Barnett, GC 748

Barranger, E 400

Barrett, W 1138

Bartlett, JMS 71, 1257

Bartsch, G 847

Bartsch, R 1454

Barzan, L 1580

Bassi, A 360

Basso, U 1286

Bastiaannet, E 12, 570

Bastos, DC 977

Basu, B 1797

Bathe, OF 931

Bathers, S 1257

Batra, SK 501

Bauer, J 422

Baumann, H 1534

Baxter, RC 1883

Beckmann, MW 800

Bedeir, A 1978

Bee, A 388

Beer, TM 808

Beery, E 1844

Beesley, C 388

Bégin, LR 800

Bekaert, S 1083

Belaj, K 1244

Belge, G 1754

Bellevicine, C 626

Belotti, D 360

Ben-Av, R 814

Benchetrit, M 1944

Benedetti-Panici, P 785

Bennett, L 1554

Bensaad, K 1207

Bento, MJ 537

Beppu, T 1950

Beral, V 169, 527, 879

Berger, W 1978

Bergh, J 18

Berghoff, AS 1454

Bergkvist, L 874

Berkers, J 1665

Bertucci, F 1433

Bessell, EM 531

Beukers, W 1392

Beumer, JH 592

Beuselinck, B 1665

Bhanot, G 1722

Bhat, AB 1618

Bhat, GA 1618

Bhatt, M 516

Bhawal, UK 700

Bhopal, SS 183

Bian, X 1488

Bianco, R 626

Biasoni, D 1227

Bidstrup, PE 201

Bielack, SS 583

Biermann, K 1153

Biernat, W 793

Bigner, DD 1481

Bijnsdorp, IV 1963

Billemont, B 455

Bindels, LB 1337

Birkeland, E 1997

Birnbaum, D 1433, 1944

Birner, P 1454

Birrell, J 255

Bischoff, J 956

Bitarte, N 287

Biti, G 308

Bitran, JD 592

Bjohle, J 18

Bjøro, T 1833

Black, A 207

Black, RJ 255

Blagden, S 925

Blaheta, RA 847

Blakely, J 2024

Blanchet, B 455

Blanco Codesido, M 1797

Blanco-Prieto, S 1876

Blay, J-Y 639

Blesing, C 585

Bloigu, R 1729

Blomqvist, C 800

Blows, F 1257, 800

Bocciolone, L 785

Body, J-J 1665

Boffetta, P 888, 1584, 1618

Bogdanova, T 994

Böhling, T 1761

Böhm, J 1761

Bojar, H 1892

Boku, N 429

Bolli, GB 1608

Bonaventura, T 588

Bond, C 1644

Bond, S 585

Boni, V 287

Boniol, M 1608

Bono, Y 448

Bordignon, C 37

Borin, M 1286

Borley, J 1069

Borras, JM 1249

Borre, M 116, 1392

Børresen-Dale, A-L 1722

Bosetti, C 1580

Bossuyt, PM 1051

Bota, M 1608

Botana-Rial, MI 1876

Bottle, A 1213

Bouchez, LC 43

Bouliotis, G 531

Boulton, ST 1481

Bourton, EC 1506

Boutros, PC 508

Bowden, SJ 1257

Bower, M 1423

Bowman, MJ 1534

Boye, K 667

Boyle, P 1608

Bradburn, DM 417

Bradley, M 531

Brakenhoff, R 1138

Bramble, MG 417

Bramhall, JL 1692

Brase, JC 1892

Brauer, K 224

Brault, L 491

Bray, IM 967, 1203

Breccia, M 904

Bremnes, R 442

Bremnes, RM 1833

Brenner, H 831

Brewster, DH 255

Briasoulis, E 75, 1423

Brinton, LA 1181

Bristow, RG 840

Britten, A 1310

Brizio, R 375

Broaddus, R 1776

Broderick, P 1001

Brodeur, GM 1418

Brodmann, M 1244

Brooks, J 100

Brophy, VH 345

Brostjan, C 961

Brown, A 1138

Brown, C 588

Brown, MD 1737

Brown, R 1069

Brown, S 1488

Browne, G 1714

Bruce, J 937

Brüning, T 1188

Brunner, A 84

Brunt, AM 1257

Brustmann, H 84

Bryan, K 967, 1203

Brydøy, M 1833

Buc Calderon, P 1337

Buda, A 785

Budczies, J 1892

Budiningsih, S 772

Bullerdiek, J 1754

Bultz, BD 617

Bulusu, R 585

Bulvick, S 1844

Bunch, KJ 1652

Burger, H 639

Burgers, JA 161

Burke, W 1093

Burkholder, I 1678

Burnet, NG 748

Burrows, FJ 707

Burt, R 925

Burton, C 1644

Büttner, P 422

Butturini, G 1883

Bützow, R 1761

Byzova, TV 713

Cairns, BJ 169, 527

Caldas, C 800, 1257

Cameron, D 71, 1257

Campagnutta, E 785

Campbell, C 243, 1938

Cani, PD 1337

Canon, J-L 435

Cappuzzo, F 793

Carbillon, L 1915

Card, TR 1602

Carey, FA 255

Carey, MS 1776

Carlomagno, C 626

Carlson, LE 617

Carmony, KC 53

Carpenter, LM 1159

Carreira, H 537

Carreon, JD 195

Carter, SA 739

Cartledge, J 1131

Carvalho, I 537

Carvalho, MA 977

Casado, A 1

Casey, G 1001

Cassinello, J 435

Castagnetti, F 904

Castanon, C 435

Castiglioni, V 360

Castro, C 537

Casula, G 675

Cataldo, D 1083

Catania, A 1978

Catto, JWF 123

Catton, C 840

Causer, PA 24

Cavalli-Björkman, N 189

Ceballos, K 1917

Celadin, R 1286

Cerny, T 266

Cesca, M 360

Cha, EK 1826

Chadwick, K 840

Chambers, AF 840

Chan, DSY 1925

Chan, S 814

Chang DZ 411

Chang, JW 1883

Chang-Claude, J 748, 831

Channathodiyil, P 1488

Charbonneau, V 556

Charlton, PA 291

Chatelut, E 1100

Chatterjee, N 207

Chattopadhyay, A 739

Cheang, M 800

Chen, J 1776

Chen, S 352

Chen, Y 1268

Chen, Y-C 2010

Cheng, A-L 1672

Chera, BS 482

Chéreau, E 401

Chevassut, TJ 1987

Chia, D 207

Chiap, P 1083

Chikamoto, A 1950

Chilcott, S 1384

Chin, X 1564

Cho, CY 43

Choi, CH 91

Choi, J-J 91

Chong, G 1864

Choudhury, J 1766

Choueiri, TK 1009

Christensen, E 840

Christensen, J 165, 201

Christner, SM 592

Christoph, DC 823

Chu, C-M 2010

Chu, LW 207

Chu, Z 352

Chua, J 1564

Chudzinski-Tavassi, AM 977

Chung, L 1883

Chung, MH 1624

Church, TR 207

Ciardiello, F 1791

Cimminiello, C 626

Citterio, G 37

Clarke, NW 1737

Clarke, SJ 695

Clements, A 243

Clerici, M 612

Clézardin, P 63

Cnattingus, S 544

Coan, A 1481

Coche, T 116

Coebergh, JW 549

Coebergh, JWW 12

Coffie, P 556

Cohen L 411

Cohen, O 1844

Cohen, RB 1268, 1277

Cohen-Armon, M 1317

Cohn, SL 1418

Coia, I 1083

Coldman, AJ 1917

Coleman, N 739, 1384

Coles, CE 748

Coletta, RD 977

Colevas, AD 604

Colizzi, F 1116

Collard, TJ 1514

Collin, F 639

Collins, GS 260

Collins, VP 462

Colombini, S 612

Comino, A 75

Concin, N 918

Cook, DA 1917

Cooper, CS 388

Coppens, K 1608

Coppola, D 1783

Coral, S 1116

Corchia, N 658

Corcoran, M 1384

Corcoran, NM 1467, 1564

Coriat, R 455

Corn, PG 1547

Cornain, S 772

Cornelio, GH 1075

Corradino, I 612

Correa, P 1584

Corrie, PG 585

Costello, AJ 1467, 1564

Cottone, F 904

Couch, FJ 800

Couch, ME 482

Coupland, VH 1908

Covre, A 1116

Cox, A 800

Coyle, B 1144

Craig, AJ 1310

Crawford, SM 1257

Creevey, L 967, 1203

Cristofanilli, M 1286

Crivelli, JJ 1826

Croci, S 1302

Crook, T 75, 375, 732, 1423

Crosby, TDL 1925

Cross, SS 800

Cummings, J 1518

Cunningham, D 435

Curado, MP 1608

Curpen, B 24

Cuzick, J 230

Czarnota, GJ 469

Czartoryska-Arłukowicz, B 793

Czene, K 18

D'Hondt, L 435

Dabis, F 556

Dahl, O 1684, 1833

Dal Pra, A 840

Dalla Santa, S 1116

Dalton, SO 201, 1901

Damery, S 243

Daneshmand, S 1826

Danforth, KN 1181

Daniel, F 585

Dar, NA 1618

Darai, E 401

Darb-Esfahani, S 1892

Darling, JL 1488

Dasoula, A 1423

Davidoff, AM 967, 1203

Davidson, S 748

Davies, E 404

Davies, JE 584

Dawsey, SM 888

de Bono, JS 1797

De Braud, F 1227

De Castro Carpeño, J 1277

de Castro, DG 1793

De Giovanni, C 1302

de Koning, HJ 778

De Luca, C 626

de Mol, BAJM 161

De Pascale, A 612

De Placido, S 626

de Preter, K 1409

De Stefani, E 1584

De Stefano, A 626

De Vincenzo, R 37

De Vuyst, H 1624

de Wijkerslooth, TR 1051

de Wit, R 1637

Dean, E 1518

Deans, DAC 143

Debruyne, P 1665

Decio, A 360

Dekker, E 1051

Delea, TE 1059

Dell' Anna, T 785

Delzenne, NM 1337

Deneo-Pellegrini, H 1584

Denkert, C 1892

Dennis, M 707

dePont Christensen, R 1169

Desjardins, A 1481

Desplenter, F 1644

Detours, V 994

Deutsch, E 308

Deux, B 63

Devi, BCR 1075

Dewulf, EM 1337

di Legge, A 37

Di Stefano, M 37

Diamandis, EP 1797

Diamantis, N 925

Diaz-Latoud, C 63

Diaz-Padilla, I 604

Dibb, M 1766

Dicheva, BM 1153

Dick, IM 1107

Dickinson, T 388

Dicks, E 800

Dieckmann, K 1454

Dieckmann, K-P 1754

Dietel, M 1892

Dietz, S 462

Dimopoulos, MA 1869

Dingemans, AMC 1820

Dittrich, C 280

Dive, C 1518

Dobbins, SE 1001

Dodson, A 388

Dohadwala, M 707

Doi, T 340, 429

Dolled-Filhart, M 2024

Dom, G 994

Dome, B 1978

Dorssers, LCJ 947

dos Santos Silva, I 18

Doughty, J 756, 864

Downing, A 1175

Draisma, G 778

Drayton, RM 123

Dubinett, SM 707

Dubois, L 508

Dubrovska, A 43

Duchnowska, R 793

Dudziec, E 123

Dufey, F 1188

Duhamel, A 1025

Dumez, H 1665

Dumont, JE 994

Dunn, JA 1257

Dunning, AM 748

Dyer, MJS 1423

Dyrskjøt, L 116, 1392

Earl, HM 1257

Eberhardt, WEE 823

Ebina, M 1745

Eckel, B 63

Eddy, D 469

Edrei, Y 658

Edwards, DR 143

Edwards, J 756, 856, 864, 1554

Efficace, F 904

Egerer, G 1840

Eggermont, AMM 1153

Eggert, A 1409

Egorin, MJ 592

Eguchi, K 1474

Ehlen, TG 1917

Eidtmann, H 956

Eigentler, TK 422

Einama, T 137

Eisen, A 24, 2005

Eisenbauer, M 1978

Eisner, F 1244

Ekouevi, DK 556

Elaidi, R 1665

Elashoff, D 707

ElguetaKarstegl, C 1423

Ellis, I 800

Ellis, P 158

Elliss-Brookes, L 1220

Elsaleh, H 1684

Elsässer, M 422

Elsberger, B 756, 864

Endo, C 1745

Engelhard, M 823

English, DR 1631

Eriksson, L 18

Ernst, E 405

Espinas, JA 1249

Estlander, A-M 1459

Esumi, H 340

Eustace, A 684

Evans, A 224

Evrard, B 1083

Ewertz, M 1901

Fabbri, M 1345

Fabi, A 793

Fairley, L-L 1608

Faison, T 1826

Falandry, C 598

Falk, RS 1451

Fankhauser, M 1564

Farrell, PJ 1423

Farrell, T 757

Fasching, PA 800, 956

Fassan, M 1286

Fassbender, K 931

Favier, L 598

Fay, J 967, 1203

Fear, NT 1652

Fearon, KCH 143

Fedier, A 1399

Fehm, T 956

Fentiman, IS 221

Fenu, E 925

Ferguson, MJ 1327

Fernández-Villar, A 1876

Fernando, I 1257

Feron, O 1337

Ferronha, I 537

Fielitz, K 1409

Filipits, M 1978

Finan, PJ 757

Finetti, P 1433

Fischer, M 1418

Fisher, D 1037

Fisher, R 1664

FitzGerald, LM 1776

Flatmark, K 667

Fleischer, F 280

Fleshner, N 840

Fletcher, CD 695

Fleuren, GJ 772, 1956

Fleury, H 556

Flor, I 1754

Fløtten, Ø 442

Fluge, O 1684

Fockens, P 1051

Foekens, JA 947

Foidart, J-M 1083

Folprecht, G 1044

Fombonne, J 63

Fonsatti, E 1116

Fonseca, E 435

Font, R 1249

Fontecha, M 230

Forbes, IWG 1554

Forbes, JF 230

Forman, D 1175

Forootan, SS 388

Fortunato, M 732

Fosså, SD 1833

Fossati, R 588, 785

Foster, CS 388

Foster, L 1257

Foster, NA 739

Foszczyńska-Kłoda, M 793

Foulkes, WD 800, 2005

Fradet, Y 1826

Franceschi, S 215, 1445, 1580, 1624

Franck, N 455

Francken, AB 570

Franco, EL 1917

Fraser, CG 255

Fratta, E 1116

Frederiksen, K 165

Freyer, G 598

Friedman, AH 1481

Friedman, E 2005

Friedman, HS 1399, 1481

Fritchie, K 482

Fritsche, H-M 1826

Froehlich, H 1244

Fruscio, R 785

Fujii, Y 1031

Fujiwara, S 1969

Fujiwara, T 448

Fukagawa, T 1345

Fukai, Y 31

Fukushima, R 724

Fukutomi, A 429

Fulp, J 1783

Fumoleau, P 598

Funkhouser, WK 482

Furukawa, H 137

Furuya, M 632

Fuse, N 340

Fushiya, N 988

Gabra, H 925

Gajewski, TF 1663

Gallego, S 1374

Gandini, S 1608

Gao, B 604

Garant, A 1864

Garavello, W 1580

Garay, T 1978

Garbe, C 422

Garcia Giron, C 435

Garcia, M 1249

Garcia-Closas, M 800

Garcia-Foncillas, J 287

Garg, MB 1525

Garmo, H 221

Garrett, CR 411

Garrigue, I 556

Garrone, O 75, 375, 732

Gary, T 1244

Gasser, M 847

Gatter, KC 1044

Gauler, TC 823

Gavalas, NG 1869

Gebski, V 588

Gehrmann, M 1892

Geldof, AA 1963

Gelmon, KA 800

Gencer, D 1678

Georgoulias, V 1932

Gerard, P 388

Ghadirian, P 2005

Ghaem-Maghami, S 1069

Ghanim, B 1978

Gherlinzoni, F 904

Giatromanolaki, A 1044

Giavazzi, R 360

Gibert, B 63

Gietema, JA 1637

Giles, A 469

Giles, G 800, 1631

Gil-Gil, M 1249

Gill, A 1883

Gill, I 158

Gill, MD 417

Gillett, C 221

Girard, E 1498

Gissler, M 544

Giurescu, M 808

Gleave, ME 1467

Glimelius, B 189

Gloesenkamp, C 1853

Goc, A 713

Godinho, MFE 947

Godoy, H 370

Gogna, R 516

Goguet-Surmenian, E 1944

Going, JJ 864

Gojis, O 75, 375, 732

Goktug, T 1488

Goldschneider, D 63

Goldwasser, F 455

Gologan, A 1864

Gomez, B 345

Gonin, V 63

Gonzalez de Castro, D 345

Gonzalez-Huarriz, M 287

Gorlia, T 639

Gorter, A 1956

Gosden, C 388

Gourley, C 1327

Grainge, MJ 1602

Graner, E 977

Grassi, A 1286

Grassi, R 785

Graubard, BI 195

Gray, K 143

Green, AR 800

Green, DA 1826

Green, J 527, 879

Green, S 2024

Greenberg, DC 800

Greenhough, A 1514

Greenslade, M 1220

Gregory, WM 1131

Greystoke, A 1518

Gridley, G 195

Grieve, R 1257

Griffiths, J 462

Grigorieva, J 1820

Grilley-Olson, JE 482

Grimm, C 918

Grinsted, L 1093

Groen, HJM 1820

Groff, SL 617

Grønberg, BH 442

Gross, E 658

Grosso, F 612

Grosso, V 1302

Grotzer, MA 1399

Grubb, R 207

Gruenberger, B 961

Gruenberger, T 961

Grundy, RG 1144

Grusch, M 1978

Gruselle, O 116

Gualberto, A 2024

Gueders, M 1083

Guichet, P-O 1488

Guillemot, E 1944

Güller, U 266

Gunawardana, NP 403

Gutin, A 1776

Haas, T 1059

Haber, M 1418

Hackman, T 482

Hackshaw, A 910, 1801

Haferkamp, A 847

Hafner, F 1244

Haga, N 31

Hagel, C 1399

Haider, S 508

Haidopoulos, D 1869

Hall, JS 684

Hall, P 18

Halloran, SP 765

Hamdy, FC 123

Hamelinck, VC 12

Hamilton, W 1205

Hammarström, M-L 150

Hammarström, S 150

Hammersley, V 1938

Hammond, EM 291

Han, N 1766

Handley, G 765

Hansen, BT 1451

Hansen, TF 1169

Harding, V 925

Hardwick, RH 1908

Hardy, R 585

Harjes, U 1207

Harper, P 158

Harrington, JA 1692

Harris, AL 1044, 1207

Harris, HR 874

Harrison, J 1125

Hart, CA 1737

Hartung, A 43

Hasegawa, M 652

Hashimoto, Y 448

Hatzidaki, D 1932

Hatzimichael, E 75, 1423

Haugland Haugen, M 667

Hauschild, M 956

Hawkins, R 435

Hayes, DN 482

Hayes, RB 207

Hayes, S 1125, 1766

Haylock, R 1660

Haynes, AP 531

Hayward, MC 482

He, C 352

Heenan, SD 1310

Hegedus, B 1978

Heijnsdijk, EAM 778

Heilmann, M 1409

Heinemann, V 1678

Heinimann, K 1399

Heinonen, H-R 1761

Heinze, G 84, 918

Henderson, BW 1534

Henderson, FC 772

Hendry, M 243

Heng, DYC 1059

Hennessy, BT 1776

Hepp, R 823

Herbst, RS 1268, 1277

Herndon, JE 1481

Herpel, E 831

Herrington, CS 1327

Herzig, K-H 1729

Heys, SD 937

Heywood, M 588

Hiki, N 275

Hill, KA 24

Hill, M 585

Hiller, L 1257

Hills, A 1138

Hiom, S 1220

Hironaka, S 429

Hirsch, FR 823

Hixon, ML 2024

Ho, Y-K 53

Hochhaus, A 1678

Hoda, MA 1978

Hodi, FS 1663

Hoekstra, HJ 570, 1637

Hoenerhoff, MJ 129

Hoffmann, B 1853

Hoffmeister, M 831

Hofheinz, R-D 1678

Hofstetter, G 918

Hofvind, S 1451

Hohenberger, P 639

Hoivik, EA 1997

Hokland, P 1761

Hollenbeck, A 1181

Hollerbach, S 280

Holmberg, L 221

Holst, F 1997

Homma, S 137

Honecker, F 1853

Hong, JT 53

Hoover, RN 408

Hop, WCJ 1153

Höpfner, M 1853

Hopper, J 1001

Hopper, JL 1631

Horenblas, S 1637

Horgan, PG 695, 856

Horiguchi, Y 652

Horne, GA 1987

Hornslien, K 442

Horo, A 556

Hoshi, E 1474

Hoskin, PJ 684

Hossfeld, DK 435

Hovens, CM 1467, 1564

Howell, A 230

Hsing, AW 207

Hsu, C 1672

Hsu, C-H 1672

Hu, D 707

Huak, CY 334

Huang, W-Y 207

Huang, X 1138

Huelsken, J 1514

Hugh, T 1883

Hughes, A 1518

Hughes, B 158

Hugnot, J-P 1488

Hugosson, J 778

Huntsman, D 800

Huo, Y 411

Huober, J 956

Hurst, C 1392

Hurt, C 243

Huson, LW 1022

Hussain, A 808

Hutchings, S 1662

Hwang, SY 108

Iacovelli, R 1227

Ianzano, ML 1302

Ide, H 652

Iezzi, M 1302

Iida, K 300

Iinuma, H 724

Ikeda, T 724

Ilhan-Mutlu, A 1454

Iliev, D 1776

Imai, N 988

Imamura, T 724

Inbar, D 1317

Inbar, O 814

Indraccolo, S 1286

Ino, K 1969

Inoue, A 1745

Inoue, M 448

Ioannou, K 1869

Iqbal, B 1618

Iqbal, J 1797, 2005

Iqbal, ST 1618

Iradji, S 469

Ireland, SJ 388

Irlam, JJ 684

Irminger-Finger, I 675

Irwin, AG 1310

Isambert, N 598

Ishibashi, M 1345

Ishibashi, T 300

Ishikawa, M 300

Ishimoto, T 1233

Ishioka, J 1031

Islami, F 888, 1618

Israelsson, A 150

Issa-Nummer, Y 956

Itoh, H 632

Iurlo, A 904

Ives, A 1220

Iwaku, A 988

Iwatsuki, M 1233, 1950

Jack, RH 1908

Jakobsen, A 1169

Jalava, T 1044

Jammulapati, S 1776

Janczar, K 1423

Jankowski, T 793

Jansen, L 831

Jaquet, A 556

Jarrard, DF 100

Jarzab, B 994

Jassem, J 793

Jeannon, J-P 1138

Jeng, W-J 2010

Jensen, M-B 1901

Jiang, X 275

Jirillo, A 1286

Jitlal, M 910

Jobsen, JJ 549

Jodrell, DI 1692

Joergensen, S 1169

Johansen, C 201

Johansson, J-E 895

Johnson, PJ 1595

Johnston, M 937

Johnston, RB 1467

Jones, C 1938

Jones, CI 1987

Jones, RJ 856

Jong, RA 24

Jordan, F 856

Jordan, L 75, 224

Jordan, SD 1257

Joshi, H 1722

Juengel, E 847

Julin, B 895

Jung, E-J 325

Kabouraki, E 1932

Kadowaki, T 2016

Kaern, J 588

Kaira, K 632

Kajiyama, H 1969

Kakhdoomi, MA 1618

Kalbakis, K 1932

Kalland, K-H 1997

Kalso, E 1459

Kamachi, H 137

Kamangar, F 888

Kamiyama, T 137

Kamo, N 724

Kämpjärvi, K 1761

Kan, L 1917

Kanai, Y 632

Kanfer, G 1844

Kannappan, V 1488

Kantola, T 1729

Kapidzic, A 1295

Kaplan, R 1037

Karadimou, A 1665

Karakiewicz, PI 1826

Karhu, T 1729

Karimdjee-Soilihi, B 1944

Karlsen, RV 201

Karn, T 956, 1892

Karp, DD 2024

Karran, LE 1423

Karrasch, M 1

Karttunen, TJ 1729

Kasai, T 1474

Kasiri, S 315

Kasper, S 823

Kassouf, W 1826

Katagiri, A 300

Katagiri, H 300

Kataja, M 1459

Kawakami, S 1031

Kawamata, F 137

Kawamoto, Y 340, 1444

Kaye, SB 1025, 1093, 1797

Ke, Y 388

Kealy, R 230

Keegan, TJ 1652

Kelly, R 1574

Kendall, E 765

Kendall, GM 1652

Kenessey, I 1978

Kenny, SL 1037

Kenter, GG 1956

Kerger, J 435

Khan, I 910, 1801

Khan, J 1418

Khandan, F 956, 1892

Khorprasert, C 1075

Khuu, A 1059

Kiemeney, LALM 1637

Kihara, K 1031

Kikkawa, F 1969

Kikuchi, E 652

Kikuchi, K 1474

Kilpivaara, O 1761

Kim, B-G 91

Kim, H 1433

Kim, H-J 91, 108

Kim, KB 53

Kim, MA 325

Kim, M-K 91

Kim, S 1268, 1277

Kim, SB 1075

Kim, T-J 91

Kim, TS 108

Kim, TY 1075

Kim, WH 325

Kim, WK 1433

Kim, Y-K 108

Kim-Sing, C 2005

King, AJ 1987

King, E 382

King, JC 1652

Kinlen, LJ 1163

Kinnersley, B 1001

Kinoshita, A 988

Kinoshita, K 1233

Kirita, T 700

Kirsh, VA 207

Kissow Lildal, T 116

Kitchen, D 158

Kitchener, H 1574

Kjaer-Frifeldt, S 1169

Kleiman, M 814

Klepetko, W 1978

Klepp, O 1833

Klikovits, T 1978

Klintrup, K 1729

Knapp, S 491

Knowles, M 1392

Knudsen, ES 1684

Kobayashi, K 1474

Kobayashi, M 1075, 1745

Kobayashi, Y 100

Köberle, D 266

Koechlin, A 1608

Koffijberg, H 161

Koga, F 1031

Koike, K 988

Koizumi, W 429

Kojima, M 340

Komatsu, Y 340

Komiyama, K 1474

Kondo, T 1745

Konfortion, J 1908

Koning, GA 1153

Kooijman, JL 772

Kool, M 1399

Kopetz, S 1442

Kordes, U 1399

Korfage, IJ 1295

Koshiol, J 207

Kosma, V-M 800

Koster, J 1399, 1409

Köster, J 1409

Kotajima, F 1474

Kotani, T 1969

Koukourakis, MI 1044

Kowoll, A 1840

Koyama, S 1474

Kozakai, N 652

Kozloff, MF 1268, 1277

Krajden, M 1917

Krakstad, C 1997

Kramar, A 1025

Kreuzer, M 1188

Krippendorff, B-F 1692

Kristeleit, RS 1025

Kristensen, V 1722

Krivak, T 1776

Kroll, ME 216, 219, 879, 1159

Kronenwett, R 1892

Kruijff, S 570

Krzakowski, M 1277

Kubota, T 275

Kuehni, CE 234

Kuester, D 675

Kuiper, JL 1820

Kuipers, EJ 1051, 1295

Kumagai, K 275

Kumaki, N 2016

Kumamoto, H 1745

Kuniyasu, H 700

Kuppusamy, P 516

Kurashige, J 1233

Kurihara, M 700

Kurosch, M 847

Kurzrock, R 1093

Kusonmano, K 1997

Kusunoki, M 1595

Kuwano, H 31

Kwast, ABG 549

Kweon, SH 108

Kyo, S 448

La Vecchia, C 1580

Labianca, R 612

Lackner, A 1978

Lacombe, D 1025

Laekeman, G 1644

Lagergren, J 564, 1908

Lahav, M 1844

Lai, D 352

Lake, RA 1107

Lamb, GW 856

Lambiase, A 37

Lambin, P 508

Lamers, C 639

Lanchbury, JS 1776

Land, LH 1901

Landi, L 793

Landis, SH 1075

Landoni, F 785

Landuzzi, L 1302

Langer, CJ 2024

Lanza, C 1286

Lao-Sirieix, S 585

Larbret, F 1944

Largillier, R 588

Larkin, J 1664

Larry Goldenberg, S 1467

Larsen, SB 201

Laskey, RA 1384

Laszlo, V 1978

Lattanzio, L 75, 375, 732, 1423

Laubender, RP 1678

Lauraine, EP 588

Laurent, D 1044

Lavelle, K 1175

Lavery, K 1138

Lawhon, T 707

Lawrence, G 1175

Lawrence, HJ 345

Lawson, EB 604

Le Cesne, A 639

Lebbe, C 455

Lebwohl, D 1044

Lee, AJ 1644

Lee, BL 325

Lee, HE 325

Lee, HS 325

Lee, J-H 1783

Lee, JM 707

Lee, J-W 91

Lee, M-H 646

Lee, S 75

Lee, SC 1075

Lee, SM 910

Lee, T-H 108

Lee, TJW 417

Lee, Y-Y 91

Lefevre, K 1644

Legood, R 1574

Leh, S 1684

Lehtonen, HJ 1761

Leighl, NB 604

Leiter, U 422

Leitzmann, MF 207

Lele, S 370

Lemoli, RM 1302

Leonard, RCF 1257

Leoni, VP 675

Lerut, E 1665

Lestingi, TM 592

Leung, HY 1554

Levine, RL 1761

Lewensohn, R 1361

Lewis, R 243

Lewis, WG 1925

Li, D 2024

Li, HK 925

Li, J 544

Li, L 675

Li, Z 1684

Lian, JB 1714

Lian, L-Y 388

Liaw, Y-F 2010

Libby, G 255

Libra, M 1580

Liedtke, C 956

Liefers, GJ 12

Lieffers, JR 931

Lieuwes, NG 508

Lightfoot, T 217

Lightsey, D 754

Lin, C-C 2010

Lin, S-M 2010

Lin, Y 707, 1692

Lin, Z-Z 1672

Lincz, LF 1525

Lind, JSW 1820

Lindebjerg, J 1169

Lindmark, G 150

Lindsay, I 1810

Linklater, KM 1908

Linnebank, M 1840

Lipp, ES 1481

Lisano, J 604

Liu, J 713, 1547

Liu, L 411, 549, 604

Liu, P 1488

Liu, S 370

Llacuachaqui, M 2005

Lo, L 1125

Lo Nigro, C 375, 732, 1423

Loesch, C 823

Loewendick, H 823

Lofts, F 435

Logan, RF 765

Logothetis, C 646, 1547

Loibl, S 956, 1892

Lollini, P-L 1302

London, WB 1418

Lone, MM 1618

Long, JS 756

Löning, T 1754

Look, MP 947

Lopez, I 287

Lopez-Rios, F 345, 1793

Lopez-Vivanco, G 435

Lord, K 1017

Lorusso, D 37

Lou, E 1481

Louwman, WJ 12, 1637

Lu, KH 1776

Lu, L-C 1672

Lu, Y 564

Luaces, ME 1584

Lubinski, J 2005

Lüchtenborg, M 221

Luk, JM 334

Lunet, N 537

Luo, J 707

Lynch, HT 2005

Lynch, J 967, 1203

Lyons, A 1138

MacCarthy, A 1652

MacDonald, K 901

Macedo, A 1938

Macheto, D 1864

MacKenzie, LM 1554

Madan, E 516

Maddams, J 1195

Madhu, B 462

Madi, A 1037

Maehara, Y 1345

Maemondo, M 1745

Maenhaut, C 994

Magen-Nativ, H 1844

Magerle, M 1454

Maggioni, A 785

Mahdi, AA 516

Maida, Y 448

Maier, T 961

Maio, M 1116

Mair, D 345

Majeed, A 1213

Makarević, J 847

Mäkelä, J 1729

Mäkinen, MJ 1729

Mäkinen, N 1761

Mælandsmo, GM 667

Malapelle, U 626

Malats, N 1392

Malekzadeh, R 888

Malki, MI 388

Mallon, E 756

Malmström, P-U 1392

Mandal, SS 315

Mandalà, M 612

Mandelli, F 904

Mangili, G 37

Mangioni, C 785

Mangoni, M 308

Mann, KD 183

Mannermaa, A 800

Manning, JT 1631

Mansi, JL 1257

Mantori, M 37

Marciano, R 626

Marfizo, S 937

Margelí, M 1249

Margulies, A-l 400

Mariadason, JM 1514

Maris, JM 1418

Marosi, C 1454

Marra, G 1399

Marreaud, S 639

Marschall, T 1409

Marsoni, S 612, 1025

Marth, C 588, 918

Martin, JC 1337

Martin, JE 477

Martin, LP 1268, 1277

Martin, M 1409

Martin, RE 1917

Martinez, R 345

Marx, A 831

Masciullo, V 37

Masià, A 1374

Masood, A 1618

Masuda, H 1031

Masuda, S 2016

Mathijssen, RHJ 1100

Matsukata, A 1239

Matsuno, Y 137

Matsuoka, Y 1031

Matsuyama, Y 1239

Matthay, KK 1418

Mau, C 956

Maughan, TS 1037

Mavroudis, D 1932

McAdam, K 585, 1257

McArdle, PA 856

McCall, P 1554

McClements, PL 255

McCormack, V 18

McCoy, MJ 1107

McGlynn, KA 195

McGurk, M 1138

McHugh, A 75

McKean, M 1384

McKenna, WG 291

McKenzie, KP 1624

McKeown, SR 1714

McLaughlin, J 1783

McLean, C 1631

McLean, D 224

McMillan, DC 695, 864

McPhail, S 1220

Meade, AM 1037

Mecklin, J-P 1761

Medani, H 925

Meehan, M 967, 1203

Mehine, M 1761

Mehlen, P 63

Mehta, MK 1892

Meier, F 422

Meijer, CJLM 1624

Meinhardt, G 1044

Meinhold-Heerlein, I 956

Mellemkjær, L 165

Menard, C 840

Mendall, MA 1908

Mendilaharsu, M 1584

Mendoza, AD 793

Meng, Z 411

Menter, T 491

Merat, S 888

Merati-Kashani, K 266

Merger, M 280

Merlano, M 75, 375, 732, 1423

Mermershtain, W 814

Messenger, MP 1131

Messner, S 24

Meyer, LA 1776

Mhawech-Fauceglia, P 370

Miah, S 123

Miatello, L 1286

Michel, G 234

Michiels, J-F 1944

Migliorini, G 1001

Miki, Y 1745

Miki, YC 1595

Milani, R 785

Miller, CJ 684

Millet, M-A 1944

Mills, GB 1776

Mills, J 748

Mills SJ 417

Milosevic, M 840

Mimori, K 1233, 1345

Min, W-S 108

Minezaki, S 1474

Ming, L 1714

Minga, A 556

Minuti, G 793

Mir, O 455

Mishra, BP 315

Misset, J-L 435

Mitchell, AJ 1017

Mitoma, Y 724

Miyajima, A 652

Miyamoto, Y 1950

Miyazaki, K 300

Miyazawa, Y 724

Mizumoto, Y 448

Mjøs, S 1997

Mochiki, E 31

Mohammed, ZMA 864

Mohri, T 1595

Mohri, Y 1595

Molife, LR 1797

Møller, H 1195, 1908

Moller, P 2005

Monclair, T 1418

Mondul, AM 1589

Montella, M 1580

Monteverde, M 75, 375, 732, 1423

Moody, AM 585

Moon, H 1075

Moons, KGM 161

Morales-Barrera, R 1025

Moreno Garcia, V 1797

Morgan, SL 123

Mori, K 1474, 1745

Mori, M 632, 1345

Morik, K 1409

Morris, EJA 757

Morris, S 765

Morrison, DH 382

Morrison, DS 575, 695

Morse, J 1595

Moser, S 925

Moshfegh, A 1361

Moskau, S 1840

Moss, S 1574

Mosseri, V 1418

Moulin, C 1

Mousseau, M 435

Mross, K 280

Muccioli, GG 1337

Mugo, NR 1624

Muirhead, CR 1660

Mukherjee, A 814

Muldrew, KL 482

Muller, DC 1631

Müller, SA 266

Müller, V 956

Mullie, P 1608

Munro, A 71, 1257

Munzert, G 280

Münzker, J 1978

Murchie, P 1644

Murgo, A 1302

Murphy, F 216, 219, 879, 1159, 1652

Murray, M 1445

Murray, RJ, S 748

Musacchio, RA 403

Myers, PH 129

Myklebust, MP 1684

Myrvold, HE 1684

Nabhan, C 592

Nabi, G 1384

Nabi, S 1618

Nagai, Y 1233

Nagamori, S 632

Naing, A 1093

Najar, MS 1618

Nakagawara, A 1418

Nakamura, M 448

Nakashima, J 652

Nakayama, K 300

Nakayama, M 1474

Nakayama, N 300

Nakayama, S 300

Namdarian, B 1564

Nanni, P 1302

Näpänkangas, J 1729

Narod, SA 24, 1783, 2005

Narumi, S 1745

Nasrollahzadeh, D 888

Natter, C 918

Naughton, M 7

Neal, DE 1384, 1467

Neal, JW 604

Neal, RD 243

Negri, E 1580

Neidle, S 584

Nemeth, C 961

Nesbitt, H 1714

Nesland, JM 667

Neuhausen, SL 2005

Neumann, D 1317

Nevanlinna, H 800, 1761

Newbury, SF 1987

Neyrinck, AM 1337

Ng, CS 411

Ng M, TH 1327

Ngan, S 925

Nguyen, F 508

Nguyen, T 1564

Niazi, T 1864

Nicholls-Elliott, H 531

Nichols, LL 1037

Nichols, PW 1204

Nickerson, C 757

Nicolay, HJMG 1116

Nicoletti, G 1302

Nicolson, M 435

Nicosia, S 1783

Nielsen, BS 1169

Nielsen, TO 800

Nigro, CL 75

Niimi, K 1969

Nikoloff, M 230

Nisar, I 1618

Nishihara, H 137

Nishino, H 988

Nitzsche, B 1853

Njoroge, JW 1624

Noel, A 1083

Noguera, R 1409, 1418

Nordenberg, J 1844

Normanno, N 1791

Novara, G 1826

Novello, S 2024

Novoradovsky, A 1392

Nowak, AK 1107

Nukiwa, T 1745

Numao, N 1031

Nunobe, S 275

Nygård, M 1451

Nyongesa-Malava, E 1624

O'Brien, M 158

O'Brien, T 793

O'Connor, S 531

O'Meara, A 967, 1203

O'Neill, KA 1652

O'Neill, R 143

O'Reilly, SM 1257

Oades, G 856

Obel, C 544

Öberg, A 150

Obermann, EC 491

Obichere, A 765

Ochiai, A 340

Oczko-Wojciechowska, M 994

Odell, E 1138

Oelmann, E 1093

Ogasawara, N 340

Ogata, K 31

Ogawa, T 632

Ogilvie, GS 1917

Ohi, Y 1239

Ohlsson, L 150

Ohnaka, S 724

Ohno, T 31

Ohotski, J 756

Ohtsu, A 340, 429

Oishi, M 988

Ojeda, B 1249

Okabe, H 1950

Okinaga, K 724

Olcina, MM 291

Olden, K 129

Olesen, F 1205

Oliphant, R 575

Oliva, P 360

Oliver, SE 1175

Olmos, D 1025, 1093

Olsen, A 165

Olsen, J 544

Olshan, AF 482

Olson, JE 800

Olszanski, AJ 1268, 1277

Omoto, D 724

Onerheim, R 1864

Ong, CW 334

Onoda, H 988

Onozawa, Y 429

Opdahl, S 176

Orange, C 756, 856, 1554

Ordóñez-Morán, P 1514

Oriuchi, N 632

Ørntoft, TF 116, 1392

Osamura, RY 2016

Osborne, M 585

Ostrowski, J 1433

Otsuki, Y 300

Ouwens, GM 1637

Oya, M 652

Oyama, T 632

Øyan, AM 1997

Páez de la Cadena, M 1876

Painter, JT 129

Pal, T 1783

Paliga, A 1864

Palladini, A 1302

Pallan, L 1595

Pallapies, D 1188

Palmieri, C 75, 375, 732

Palmqvist, R 150

Pandite, L 639

Pantaleo, MA 1433

Papoudou-Bai, A 1423

Parashar, D 585

Paraskeva, C 1514

Pardini, S 904

Paridaens, R 1665

Parisi, G 1116

Park, Y 1181

Park, Y-A 91

Park, YH 1075

Parnell, J 1506

Parpinel, M 1580

Parris, CN 1506

Parsons, C 1213

Partridge, M 1138

Passaperuma, K 24

Pasterfield, D 243

Pasterk, M 1608

Patel, MR 482

Patel, N 1506

Paterson-Brown, S 143

Pati, U 516

Patnick, J 243, 757, 1574

Paulissen, G 1083

Paz-Ares, LG 2024

Peacock, S 1917

Pearce, MS 183

Pearson, ADJ 1418

Pécuchet, N 455

Pedersen, J 1564

Peglow, A 823

Pejovic, T 370

Peleteiro, B 537

Pels, H 1840

Pelttari, LM 1761

Pena Murillo, C 1138

Penel, N 1025

Peng, Y 382

Penninckx, B 1

Pennington, CJ 143

Perera, M 1384

Peres, A 400

Perisanidis, B 961

Persson, EC 195

Pesenti, E 360

Pestell, RG 1684

Peters, AAW 772

Peters, KB 1481

Peters, U 207

Petersen, K 1997

Peto, J 406

Petricoin, EF 947

Pett, MR 739

Pfeiffer, P 189

Pfister, C 1702

Pflugfelder, A 422

Pfrommer, H 1702

Pharoah, P 800, 1257

Philip, PA 1354

Piccirillo, MC 588

Piccirillo, SGM 462

Pichler, M 1244

Pietragalla, A 37

Pietsch, T 1399

Pilger, E 1244

Pilyugin, M 675

Ping, S 2005

Pintilie, M 840

Pinto, C 1791

Pires, IM 291

Pirie, K 879

Plewes, D 24

Ploner, A 18

Ploquin, A 1025

Plowman, PN 1506

Poettgen, C 823

Pogoda, JM 1204

Polesel, J 1580

Politi, E 1869

Polkowski, M 1433

Pollak, MN 2024

Pollard, JR 291

Polterauer, S 918

Poole, CJ 1257

Popanda, O 748

Porcu, L 1227

Porporato, P 1337

Potter, J 1776

Pötter, R 918

Pourshams, A 888

Powell, R 937

Pradelli, E 1944

Prat, A 1249

Preston-Martin, S 1204

Preusser, M 1454

Price, SJ 462

Pritchard, S 1125

Proby, C 75

Procopio, G 1227

Proctor, MJ 695

Provenzano, E 1257

Provenzano, E 800

Puglisi, M 1797

Puisset, F 1100

Punt, S 1956

Purdie, C 224

Purwoto, G 772

Pycha, A 1826

Pylkäs, K 800

Pyne, NJ 756

Pyne, S 756

Qayyum, T 856

Qiao, W 1547

Quéva, C 1498

Quinlan, P 75

Quirke, P 757

Quraishi, SM 195

Qvortrup, C 189

Rachiglio, A 1791

Rack, B 956

Rado, T 1268, 1277

Raeder, MB 1997

Rafiq, R 1618

Raghavan, D 1017

Ragone, A 2005

Rahardja, D 382

Rahman, M 300

Rahman, MT 300

Rahmann, S 1409

Rai, B 1384

Rai, Y 1239

Rainbow, S 765

Raine, R 765

Rajamanickam, V 100

Rakha, E 800

Ram, R 1844

Ramsay, EA 24

Rana, D 1384

Rana, FS 1624

Ranson, M 1518

Ray, DM 129

Raymond, E 1093

Razak, ARA 604

Rea, DW 1257

Real, FX 1392

Reaper, PM 291

Reardon, DA 1481

Rebholz, CE 234

Reddy, VA 43

Reed, N 1

Rees, C 417, 757

Reeves, GK 169, 527, 879

Reichmann, H 1840

Reid, TD 1925

Reijerink, JCIY 1295

Reinert, T 116, 1392

Reinthaller, A 918

Reiter, M 847

Renehan, AG 1518

Reventós, J 1374

Rich, JN 1481

Richards, FM 1692

Richards, M 1220

Riddick, ACP 143

Rink, M 1826

Risch, HA 1783

Risk, JM 388

Riss, P 84

Rixe, O 435

Rizzo, A 1116

Ro, J 1075

Roberts, I 739

Roberts, JD 129

Roberts, SA 1925

Robertson, A 1692

Robertson, C 1608

Rocks, N 1083

Roder, H 1820

Roder, J 1820

Rodolakis, A 1869

Rodriguez, J 287

Rodriguez, R 435

Rodríguez-Berrocal, FJ 1876

Rody, A 1892

Rogers, HA 1144

Rogiers, A 1665

Rohatgi, N 158

Rolke, H 442

Roma, C 1791

Roma, J 1374

Roman, E 217

Romani, F 1302

Romundstad, PR 176

Roncin, L 556

Ronco, AL 1584

Rosario, DJ 123

Rosbrook, B 1268, 1277

Rosen, B 1783

Rosenstock, J 1608

Roser, F 1702

Ross, JA 143

Ross, RK 1204

Rossi, AR 904

Rossi, E 793, 1286

Rosti, G 904

Roth, K 808

Roth, W 831

Roukema, JA 549

Roy, A 435

Rozendaal, L 1963

Rudolph, A 831

Rudraraju, B 375, 732

Rueegg, CS 234

Rugge, M 1286

Rui, H 1684

Rumbles, S 158

Rushton, L 1662

Rutkowski, S 1399

Rutter, MD 757

Ryan, J 967, 1203

Sadanandam, A 501

Sadique, Z 1574

Saeb-Parsy, K 1384

Sagara, Y 1239

Sahin, E 1978

Saito, K 1031

Saito, S 1233

Sakamoto, Y 1950

Sakoda, LC 207

Sakoff, JA 1525

Sakr, SR 1624

Sakura, M 1031

Salatiello, M 626

Saleh, H 1354

Salim, H 1361

Salto, C 1144

Salto-Tellez, M 334

Salvesen, HB 1997

Salvucci, M 904

Samol, J 1310

Samonigg, H 1244

Sampson, JH 1481

Samra, J 1883

Samrao, D 370

Samuel, TA 1268, 1277

San Pedro-Salcedo, M 604

Sánchez de Toledo, J 1374

Sánchez-Otero, N 1876

Sandstad, B 667

Sanfilippo, R 612

Sano, T 275

Sansom, OJ 1554

Saridaki, Z 1932

Sarkar, FH 1354

Sarker, S-J 1801

Sarode, VR 382

Sasahira, T 700

Sasaki, M 1239

Sasaki, T 429

Sasako, M 1345

Sasano, H 1745

Sasco, AJ 556

Sasieni, P 243

Sathornsumetee, S 1481

Sato, I 1745

Saunders, MP 1518

Sava, T 1286

Savage, PM 1810

Scambia, G 37, 785

Scanziani, E 360

Scarpini, C 1384

Scarpini, CG 739

Schaapveld, M 570, 1637

Schackert, G 1840

Schalhorn, A 435

Schauer, D 961

Schelch, K 1978

Schellens, JH 1025

Schem, C 956

Scherr, DS 1826

Scheulen, ME 280

Schildkraut, J 1783

Schlegel, U 1840

Schleiermacher, G 1418

Schmatloch, S 956

Schmid, P 1423

Schmid, RM 280

Schmid-Alliana, A 1944

Schmid-Antomarchi, H 1944

Schmidt, MK 800

Schmidt-Wolf, IGH 1840

Schmied, BM 266

Schneider-Stock, R 675

Schnelzer, M 1188

Schöffski, P 435, 1025, 1665

Schowe, B 1409

Schrader, M 1853

Schramm, A 1409

Schrewe, H 1761

Schuler, M 823

Schulte, JH 1409

Schultz, PG 43

Schutte, J 280

Schwaller, J 491

Schwartz, G 1093

Scorgie, FE 1525

Scott, KP 1337

Scott, NW 937

Scurr, M 639

Sebire, NJ 1810

Sebjørnsen, S 189

Segawa, A 632

Segersten, U 1392

Segui, MA 1249

Seguin, F 977

Seibold, P 748

Selby, PJ 1131

Sellers, TA 1783

Semmler, A 1840

Semnani, S 888

Sendur, MAN 1815

Senter, L 2005

Serraino, D 1580

Sestak, I 230

Sethi, S 1354

Setinek, U 1978

Severi, G 800, 1631

Seymour, MT 1037

Seynhaeve, ALB 1153

Seywright, M 856, 1554

Shaboodien, R 925

Shafi, R 1618

Shafique, K 575

Shah, IA 1618

Shah, K 1797

Shah, N 1467

Shah, R 1423

Shah, SA 1618

Shakeri, R 888

Shao, Y-Y 1672

Shariat, SF 1826

Sharma, N 1467

Sharrocks, AD 1766

Shaw, P 1783

Shebl, FM 207

Shelton, J 1220

Shen, Y 411

Shen, Y-C 1672

Shepherd, FA 604

Shepherd, L 1327

Sherman, M 800, 1181

Sherriff, M 1138

Shi, MM 1044

Shibata, K 1969

Shieh, F 345

Shigematsu, N 652

Shilo, N 1844

Shimizu, K 632

Shimomoto, T 700

Shimwell, NJ 1595

Shinjo, K 1969

Shockley, WW 482

Shores, CG 482

Short, D 1810

Shumak, R 24

Sidhu, SS 501

Siesling, S 549

Sieuwerts, AM 947

Sigalotti, L 1116

Signorelli, M 785

Silva, C 1584

Sim, SH 1131

Simmonds, P 1257

Simoens, S 1644

Simon, AE 1938

Simon, R 1997

Singer, CF 2005

Singh, RK 501

Singh, S 501

Sipilä, R 1459

Sita-Lumsden, A 1810

Siu, LL 604

Sivridis, E 1044

Skipworth, RJE 143

Sleijfer, S 639, 947

Smans, M 1608

Smartt, HJM 1514

Smit, EF 1820

Smith, A 217

Smith, DC 808

Smith, G 1327

Smith, LW 1917

Smith, P 388

Smith, RC 1883

Smith, S 765

Smith-McCune, K 1776

Snijders, PJF 1624

Snowball, J 765

Soerensen, FB 1169

Soerjomataram, I 549

Sofroni, E 469

Sogl, M 1188

Sohn, JH 1075

Soljak, M 1213

Som, A 646, 1547

Som, R 403

Somanath, PR 713

Song, JH 108

Song, SY 91

Song, T 91

Sonpavde, G 1009

Sonveaux, P 1337

Sörbye, H 189, 435

Sørensen, TIA 165

Sorge, J 1392

Soria, J-C 1025

Sotoudeh, M 888

Souglakos, J 1932

Soulières, D 1059

Sousa, S 1144

Soussan-Lazard, K 598

Southey, M 800

Span, PN 508

Sparreboom, A 1100

Spatz, A 1864

Speirs, R 1564

Speleman, F 1409

Spelman, AR 411

Spiekermann, M 1754

Spooner, DA 1257

Spycher, BD 234

Srivastava, S 334

St John, MA 707

Stallings, RL 967, 1203

Stamatis, G 823

Stanley, A 1257

Starlinger, P 961

Starmans, MHW 508

Starr, A 1268

Stebbing, J 158, 1423

Steele, RJC 255

Steffen, T 266

Steger, GG 1454

Steidl, K 1244

Stein, GS 1714

Stewart, GD 143

Stewart, HJS 1987

Stewart, K 1384

Stiller, CA 216, 219, 1159

Stintzing, S 1678

Stivani, V 1302

Stöger, H 1244

Stoker, J 1051

Stokes, A 143

Stoop, EM 1051

Straif, K 1188

Stram, DO 1204

Stramucci, L 1302

Strathdee, C 1498

Strumberg, D 280

Stuart, GCE 1917

Sturdza, A 918

Suarez-Gauthier, A 345

Sumrall, AL 1481

Sun, J 352

Sun, M 1826

Sun, P 1783

Sun, W 482

Sunaga, N 632

Sundstrøm, S 442

Sung, CO 91

Sunose, Y 632

Surya, IGD 772

Susanto, H 772

Sutandyo, N 1075

Sutani, A 1474

Suzuki, E 652

Suzuki, T 1745

Svatek, RS 1826

Sweep, FCGJ 508

Sweetland, S 169

Syed, N 75, 375, 732, 1423

Symonds, RP 748, 1017

Szostakiewicz, B 793

Taeger, D 1188

Tagawa, ST 1826

Taghavi, S 1978

Tahara, M 340

Tajiri, H 988

Takahashi, K 137

Takahashi, M 340

Takahashi, N 137

Takahashi, Y 1345

Takakura, M 448

Takayanagi, N 1474

Takekoshi, S 2016

Taketomi, A 137

Takeyoshi, I 632

Talamini, R 1580

Talbot, CJ 748

Tamada, S 1239

Tamandl, D 961

Tamura, J 724

Tanaka, S 137

Tang, X 2016

Taniguchi, M 137

Tanimoto, A 1239

Tanteles, GA 748

Tarabichi, M 994

Tarantino, I 266

Tarazi, J 1268, 1277

Tasmuth, T 1459

Tatagiba, MS 1702

Tawadros, T 1737

Taylor, DG 584

Taylor, J 684

Taylor, R 1093

Teh, M 334

Tempińska-Szałach, A 793

ten Hagen, TLM 1153

Tenet, V 1624

Teng, M 1595

Terpos, E 1869

Testa, I 1227

Thavasu, P 1797

Theegarten, D 823

Thibault, A 604

Thomakos, N 1869

Thomas, DC 1204

Thomas, G 994

Thomas, JD 757, 1175

Thomas, JS 71

Thommen, S 491

Thompson, A 75, 224, 732, 937, 1423

Thompson, D 1131

Thompson, Z 1783

Thoms, JW 840

Thomson, K 224

Thorne, L 482

Thornton, AJ 937

Thottakam, B 1384

Timms, KM 1776

Tiribelli, M 904

Tivnan, A 967, 1203

Tjønneland, A 165, 201

Tod, M 455

Todo, S 137

Tokuda, Y 2016

Tolzien, K 592

Tominaga, H 632

Tonini, GP 1418

Torán, N 1374

Török, S 1978

Tortorici, M 1268, 1277

Toth, C 831

Toure, B 556

Toyomasu, Y 31

Trabert, B 1181

Tracey, L 1203

Tracy, EC 1534

Tran, TH 1684

Tran, TV 1776

Tran, WT 469

Trarbach, T 1044

Travis, RC 527

Trillet-Lenoir, V 598

Trimbos, JB 772, 1956

Trinh, Q-D 1826

Trinkaus, K 7

Troiani, L 1286

Troncone, G 626

Trovik, J 1997

Trueblood, E 1498

Truong, M 100

Tsang, C 1213

Tsaur, I 847

Tsiatas, M 1869

Tsitsilonis, O 1869

Tsu, V 1445

Tsuchihara, K 340, 1444

Tsuchimochi, S 1239

Tu, B 646

Tu, S-M 646, 1547

Tucker, O 1595

Tung, N 2005

Tuomisto, A 1729

Turner, S 1481

Tusquets, I 1249

Tuthill, M 925

Tutt, A 221

Tveit, KM 1684

Twelves, C 71, 1025, 1257

Tzankov, A 491

Uchida, N 31

Ueda, N 700

Ueo, H 1345

Ulus-Senguloglu, G 1506

Umekita, Y 1239

Umezawa, K 652

Umezu, T 1969

Underwood, MA 1554

Unger, K 994

Untch, M 956

Urata, Y 448

Urch, CE 925

Utley, M 1195

Uziel, O 1844

Vaculova, AH 1361

Vahteristo, P 1761

Vainas, O 814

Vainstein, V 814

Valdez, TV 592

Valentine, H 684, 1125, 1766

Valentini, F 1608

Vallier, A-L 1257

Vamvakas, L 1932

van Agthoven, T 947

van Ballegooijen, M 1295

Van Cann, T 1665

van Dam, L 1295

van de Vijver, MJ 161

Van de Voorde, WM 1

van den Belt-Dusebout, AW 1637

van der Aa, M 570

van der Bij, S 161

van der Most, RG 1107

van der Sangen, M 12

van der Zee, JA 1153

van Eijck, CHJ 1153

Van Heugen, J-C 1083

van Laarhoven, HW 508

van Leerdam, ME 1051, 1295

van Leeuwen, FE 1637

van Moorselaar, RJA 1963

van Niekerk, DJ 1917

Van Poppel, H 1665

van Roon, AHC 1295

van't, Veer, LJ 800

Vandesompele, J 1409

Vardakis, N 1932

Varella-Garcia, M 793

Varney, ML 501

Vasudev, NS 1131

Vatten, LJ 176

Vauthey, J-N 1442

Väyrynen, JP 1729

Vázquez-Iglesias, L 1876

Velasco, P 1374

Velez, M 345

Vergote, I 588

Verity, L 24

Vermeulen, J 1409

Verrax, J 1337

Versteeg, R 1409

Verweij, J 1025

Verzoni, E 1227

Vestergaard, M 544

Vet JNI 772

Vichapat, V 221

Vignetti, M 904

Viguier, M 455

Viktorsson, K 1361

Villines, D 592

Vincent, TJ 1652

Vinnicombe, S 224

Virtamo, J 1589

Vitalini, C 612

Vitfell Pedersen, J 1797

Vivenza, D 75, 1423

Vlahos, G 1869

Voest, EE 1025

Vogt-Schaden, M 1840

von Bueren, AO 1399

von der Weid, NX 234

von Minckwitz, G 956, 1892

von Plessen, C 442

von Wagner, C 765, 1938

von Weikersthal, LF 1678

Vonen, B 1684

Voogd, AC 12, 549

Vornanen, J 1729

Voutsina, A 1932

Vozenin, M-C 308

Vredenburgh, JJ 1481

Vuong, T 1864

Waaga-Gasser, A-M 847

Wagner, J 100

Wagner, U 588

Wakelee, HA 604

Walker, AJ 1602

Walker, JR 43

Waller, A 617

Waller, J 1938

Walsh, L 1188

Wang, D 370

Wang, G 707

Wang, H 75

Wang, J 352, 808

Wang, W 1488

Wang, X 1547

Wang, X-S 527

Wang, Y 808

Ward, DG 1595

Wardle, J 765, 1938

Warner, E 24

Warren, AY 1467

Warschkow, R 266

Wasan, H 1037

Watanabe, M 1233, 1950

Watanabe, T 724

Watson, M 1644

Watts, C 462

Wedel, S 847

Wehenkel, M 53

Wei, W 1595

Weide, B 422

Weilbaecher, K 7

Weinstein, SJ 1589

Weiss, C 1844

Weiss, E 1892

Weissler, MC 482

Welch, I 1125

Weller, D 243, 1938

Wells, M 937

Welter, S 823

Welzel, TM 195

Wentzel-Larsen, T 189, 442, 1833

Wentzensen, N 1181

Werner, HMJ 1997

West, C 684, 748, 1125, 1766

Wever, EM 778

Whelehan, P 224

Whiffin, N 1001

Whitehouse, LE 757

White-Koning, ML 1100

Widhalm, G 1454

Wiesner, C 847

Wik, E 1997

Wilhelm-Benartzi, C 1069

Wilkerson, MD 482

Wilkinson, C 243

Wilkinson, JR 757

Willems, L 1665

Williams, AC 1514

Williams, D 1776

Williams, GT 1925

Wilson, A 1384

Wilson, CB 585

Wilson, G 585

Wilson, RH 1037

Wilson, S 243

Wind, TC 1131

Winget, M 931

Winqvist, R 800

Wishart, GC 800

Wist, EA 1833

Witjes, JA 1637

Witte, M 422

Witzel, I 956

Wohlschlaeger, J 823

Wolach, O 1844

Wolf, CR 1327

Wolk, A 874, 895

Wolk, M 477

Wolter, P 1665

Wong, CKE 1564

Wong, DWY 1075

Wong, JW 24

Wong, KF 334

Wong, L-M 1467

Worni, M 266

Worthington, J 1714

Wouters, BG 508

Wright, FC 24

Wulfkuhle, JD 947

Wullschlegar, S 501

Xiao, Z 352

Xue, A 1883

Xylinas, E 1826

Yaffe, MJ 24

Yamada-Okabe, H 1745

Yamaguchi, T 275

Yamamoto, E 1969

Yamamoto, K 700

Yang, B 100

Yang, C-S 501

Yang, H-K 325

Yang, HP 1181

Yang, P 411

Yang, TYO 169

Yao, S 388

Yap, YS 1075

Yarnold, JR 748

Ye, H 352

Ye, W 888

Yeoh, EM 1075

Yeoh, KG 334

Yeoh, K-W 404

Yeung, S-CJ 646

Yin, X-Y 482

Yokoyama, M 1031

Yoshimura, S 2016

Yoshino, T 340, 429, 1444

Yotsumoto, D 1239

You, B 598

Zabolotskaya, MV 1987

Zahm, DM 1892

Zaknoen, SL 707

Zamarchi, R 1286

Zanation, AM 482

Zanetta, S 598

Zarate, R 287

Zargar, SA 1618

Zauber, AG 1295

Zecchin, KG 977

Zengerling, F 1853

Zengin, N 1815

Zerbib, M 1826

Zhang, J 352

Zhang, X 448

Zhang, Y 1608

Zhang, Y-Q 675

Zhao, N 482

Zhao, Q 411

Zheleva, D 1692

Zheng, T 1608

Zhivotovsky, B 1361

Zhong, L 617

Zhou, C 207

Zieger, K 116

Zielinski, CC 1454

Zijlmans, HJMAA 1956

Zilio, F 1286

Ziogas, AC 1869

Ziti-Ljajic, S 598

Zlotta, AR 123

Zok, J 793

Zong, D 1361

Zorcolo, L 675

zu Eulenburg, C 956

Zuiverloon, TCM 1392

Zuziak, D 793

Zwarthoff, E 1392

